# Perception and Adoption of Good Agricultural Practices Among Family Farmers Supplying Fruits and Vegetables to Brazil’s School Feeding Program—A Mix-Method Study in the Federal District

**DOI:** 10.3390/foods15071225

**Published:** 2026-04-03

**Authors:** Isabela C. C. Alves, Hevellyn S. Silvestre, Amanda B. Costa, Matheus R. Driessen, Neusa K. F. Mathias, Letícia P. Souza, Sueny A. Batista, Eleuza R. Machado, Renata Puppin Zandonadi, Veronica C. Ginani

**Affiliations:** 1 Department of Nutrition, Faculty of Health Science, University of Brasília, P.O. Box 4569, Brasília 70904-970, DF, Brazil; isabelaalves11@gmail.com (I.C.C.A.); hevellynsilvestre.nutri@gmail.com (H.S.S.); amandaborbaac@gmail.com (A.B.C.); dries3012@gmail.com (M.R.D.); karolineneusa032@gmail.com (N.K.F.M.); leticiapereirasouzaa70@gmail.com (L.P.S.); renatapz@unb.br (R.P.Z.); 2 Faculty of Nutrition, Federal University of Goiás, Rua 227, Quadra 68, nº30, Setor Leste Universitário, Goiânia 74605-080, GO, Brazil; subatistanutricionista@gmail.com; 3 Hospital Universitário de Brasília (HUB), Brazilian Company of Hospital Services (EBSERH), SGAN 605, Asa Norte, Brasília 70840-901, DF, Brazil; eleuzarodriguesmachado498@gmail.com

**Keywords:** good agricultural practices, family farming, school feeding, rural health, public policy

## Abstract

To assess food safety conditions among family farmers supplying the National School Feeding Program (PNAE) in the Federal District, Brazil. This exploratory mixed-methods study was subdivided into two main phases: (i) samples of fruits, vegetables, water, soil, and farmers’ feces were analyzed microbiologically and/or parasitologically across nine properties; (ii) sociodemographic and Good Agricultural Practices (GAP) questionnaires were administered, followed by semi-structured interviews to evaluate their perceptions of food safety. Participants were males (100%), of mixed race (88.9%), aged 41–50 years (44.4%), with secondary education (33.3%), and an income between USD 1000 and USD 2000 (33.3%). Samples from food (n = 162), water (n = 18), soil (n = 90), and feces (n = 6) were analyzed. All fruit and vegetable samples, and 83.3% of water samples exceeded acceptable limits for at least one of the microorganisms analyzed. 86.7% of the soil samples showed high levels of contamination. Parasitic contamination was detected in 50.6% of the fruit and vegetable samples, in 63.3% of the soil samples, and in none of the water samples. Most farms used deep or artesian wells (77.7%) and non-connected septic pits (77.7%). Organic fertilization predominated (88.8%), with chemical fertilizers occasionally used (11.2%). Farmers demonstrated strong environmental awareness but limited technical knowledge of food safety. Results indicate persistent vulnerability despite ethical and ecological commitment. Continuous training and stronger public policies are essential to enhance GAP adherence, ensure food microbiological safety, and sustain PNAE objectives.

## 1. Introduction

Family farming plays a central role in sustainable food systems, especially in contexts of promoting food security, environmental conservation, and territorial development. It contributes decisively to the diversification of production, the reduction of dependence on external inputs, the maintenance of agricultural biodiversity, and the generation of local income, in addition to strengthening social and cultural ties in rural areas [1]. In Brazil, the National School Feeding Program (PNAE) amplifies this relevance. It functions as a structuring instrument for public policy by guaranteeing a stable institutional market for family farmers, stimulating more sustainable production practices, and promoting students’ access to fresh and culturally appropriate foods [2].

The procurement of food from family farming for school meals is an effective strategy to improve the quality of students’ diets. It also strengthens local agricultural production and fosters regional development. Shortening the food supply chain contributes to the availability of fresh, local products and helps establish healthy food preferences among students. Consequently, this practice supports food security and the realization of the Human Right to Adequate Food (HRAF) within schools [3,4].

This initiative aligns with Sustainable Development Goal (SDG) 2, which aims to eradicate hunger, achieve food security, and promote sustainable agriculture. In this regard, the adoption of Good Agricultural Practices (GAP) is a strategic pillar for the sustainability of agri-food systems, integrating environmental, sanitary, social, and economic dimensions. GAP constitutes a set of practices aimed at reducing risks to human health, protecting the environment, ensuring decent working conditions, and guaranteeing the economic viability of production. These practices range from soil and water management, input and waste control, worker hygiene and health, to care in harvesting, post-harvesting, and marketing. They should be understood as an integrated system, in which effectiveness depends on the articulation among its different blocks, rather than on the isolated adoption of environmental or productive practices, and is directly aligned with the principles of food safety and the One Health approach [5,6].

Accordingly, it is crucial to conduct a rigorous assessment of the quality of products destined for school meals, from cultivation to consumption. The adoption of control measures, such as the application of GAP in accordance with sanitary regulations, helps ensure food safety for students and prevents outbreaks of foodborne diseases (FBD) [7,8].

The exclusive supply of fruits and vegetables from family farming to public schools in the Brazilian Federal District (FD) began in 2019. At the same time, the agricultural area within the FD has expanded, enhancing the connection between local production and school feeding [4]. However, information on the hygienic–sanitary quality of foods produced by family farmers in the FD after this policy change remains scarce, particularly within the school feeding context. Furthermore, little is known about farmers’ perceptions of GAP adoption [9,10].

Therefore, this study aimed to assess food safety conditions among family farmers supplying the National School Feeding Program (PNAE) in the Federal District, Brazil, by integrating microbiological and parasitological analyses (foods, water, soil, and farmers’ feces) with the evaluation of GAP and farmers’ perceptions. Understanding the various aspects related to food safety in the context of family farming is thus essential. Analyzing farmers’ perceptions of their daily practices in implementing GAP can enable impactful public health actions for the local population, particularly within the school community. The findings may guide more effective educational and risk-prevention strategies in rural areas, thereby contributing to the reduction of FBD outbreaks.

## 2. Materials and Methods

This is a mixed-methods study, approved by the Research Ethics Committee of the Faculty of Food Sciences at the University of Brasília (CAEE n° 02033218.0.0000.0030). Inclusion criteria for selecting the properties were defined based on their owners. Therefore, the inclusion criteria were the following: (i) Be a supplier of vegetables and fruits to the PNAE in the Federal District, Brazil; (ii) Be a member of the cooperatives/associations indicated by EMATER—DF; (iii) Be nominated by the cooperative/association to which they are affiliated.

The Mixed Methods Appraisal Tool criteria were used for the study design. Thus, the mixed-methods research was developed to integrate the interviewees’ reports with sociodemographic data and microbiological and parasitological results. The lack of integration of this information would likely prevent an adequate interpretation of family farmers’ perceptions of GAP. The information therefore has both complementary and sequential characteristics as observed in the results and discussion sections.

The study’s structure was conceived based on three previously defined hypotheses.

**Hypothesis** **1 (H1).** *Owners (farmers) in vulnerable situation have difficulty adhering to GAP, increasing the risk of food contamination.*

**Hypothesis** **2 (H2).** *Properties with poor sanitation, inadequate waste disposal, and deficient sanitary facilities are unlikely to adopt GAP.*

**Hypothesis** **3 (H3).** *Failure to adhere to GAP is a determining factor in the presence of high levels of microbiological and parasitological contaminants in soil, water, and food.*

Thus, H1 concerns sociodemographic factors that can affect access to information, investment capacity in the property, and participation in training and adherence to GAP. H2 was established based on structural determinants of the property, with effects on different aspects. The main ones are: (i) personal and environmental hygiene; (ii) infrastructure necessary for pre- and post-harvest food handling; and (iii) vector and pest control. These determinants would be prerequisites for adopting GAP. The GAP would be the main limiting factor for the sanitary quality of the food produced on the property. Microbiological and parasitological analyses will indicate whether the relationships between the responses presented and the realities experienced on each property are compatible with the established parameters.

**Figure 1:**
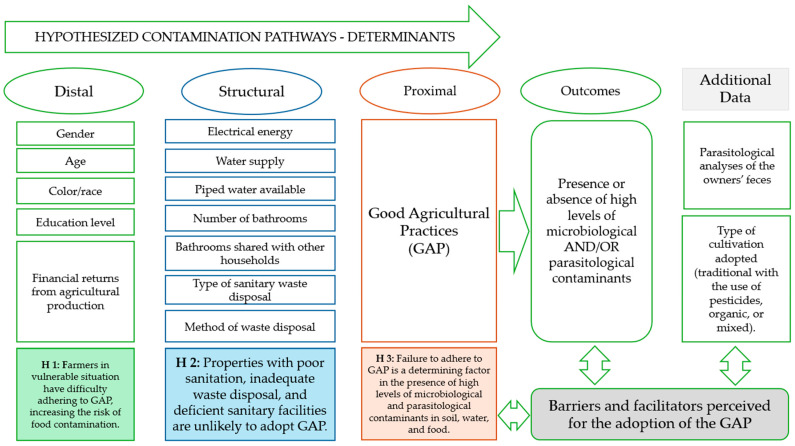
Distal-to-proximal framework of hypothesized determinants of contamination pathways on family farms, including potential outcomes, complementary data sources, and their interaction with perceived barriers and facilitators to the adoption of Good Agricultural Practices (GAP) among family farmers supplying the National School Feeding Program (PNAE), Federal District, Brazil.

Furthermore, parasitological analyses of the owners’ feces were used as indicators of human exposure and possible involvement in shared fecal-oral transmission cycles. Information on the type of cultivation adopted (traditional with the use of pesticides, organic, or mixed) was used as a potential differentiator of the *modus operandi* of one property from another. Semi-structured interviews were used to complement the model, exploring the perceived barriers and facilitators to the adoption of the GAP (Figure 1).

The study was divided into four stages: (i) participant selection; (ii) on-site data and sample collection; (iii) laboratory analyses; and (iv) data analysis. These stages were detailed below.

### 2.1. Sample Selection

The School Food Management Department of the State Secretariat of Education of the Federal District (SEE-DF) was contacted to arrange a meeting between the research team and partners from the Technical Assistance and Rural Extension Company of the Federal District (EMATER—DF). As agreed, EMATER staff invited representatives from the cooperatives/associations responsible for supplying school meals to a meeting.

At this meeting, the project was presented, and representatives from the six cooperatives and associations committed to disseminating the research among farmers and to seeking out family farmers interested in participating, composing a convenience sample. Each cooperative was to nominate at least three farmers. Once the participation of the nominated individuals was confirmed and they agreed to participate by signing the consent form, visits to their properties were scheduled.

### 2.2. Data and Sample Collection

Visits to the properties for sample and data collection took place from December 2023 to January 2024. Samples of different fruits and vegetables, water, soil, and feces from the corresponding farmers were collected for analysis. In addition, two distinct questionnaires were administered: one to identify sociodemographic characteristics and another specifically to evaluate GAP, followed by interviews on farmers’ perceptions of these practices. Each data collection action will be described separately.

#### 2.2.1. Fruit and Vegetable Sampling

Samples of fruits and vegetables were collected directly from cultivation areas, following standardized sampling procedures for microbiological and parasitological food analysis [11,12]. A total of three food items from each property were sampled according to seasonal availability, including leafy vegetables, roots, and fruits commonly supplied to school meals. Each food item was collected in triplicate. During collection, researchers wore disposable gloves, and samples were placed in sterile plastic bags, properly labeled, and immediately stored in insulated thermal boxes containing reusable ice packs. All samples were transported to the Laboratory of Microbiology and Food Hygiene at the University of Brasília and stored under refrigeration (4 °C) for a maximum of 24 h before analysis, as recommended for fresh produce [11,12].

#### 2.2.2. Water Sampling

Water samples were collected at one sampling point per property, corresponding to the water source used for irrigation and pre-washing of food, following the protocol described by Midura and Bryant [11]. Before collection, the internal and external surfaces of the taps were disinfected with 70% ethanol and flamed to minimize external contamination. Sterile 500 mL glass bottles were used for sampling. The samples were transported to the laboratory under refrigerated (4 °C) conditions [12].

#### 2.2.3. Soil Sampling

Soil samples were collected from five different points in each property: four at the margins of the cultivation area and one at the center. Samples were obtained from the surface layer (0–5 cm) and from approximately 15 cm depth using a metal spatula. After each collection, the spatula was disinfected with 70% ethanol to prevent cross-contamination. Approximately 250 g of soil from each sampling point was placed in sterile plastic bags, labeled, and transported to the laboratory for microbiological analyses and the other half for parasitological analyses.

#### 2.2.4. Feces

Fecal samples were collected exclusively from family farmers responsible for the property who agreed to participate in the study. Sterile, labeled collection containers were delivered in person at each property, along with standardized verbal and written instructions regarding proper sample collection to avoid environmental contamination. Approximately 2 g of fecal material was collected from each participant. Samples were returned to the research team under refrigeration and processed within 24 h at most.

#### 2.2.5. Application of Questionnaires and Interviews with the Owners

##### Sociodemographic Characteristics and Adoption of Good Agricultural Practices

The person responsible for each property answered a questionnaire for sociodemographic characterization (Part 1) (gender, age group, color/race, education level, and financial returns from agricultural production) and of the property infrastructure (Part 2) (presence of electricity, condition of sanitary facilities, waste management, basic sanitation of the property) [13,14]. In addition, questions were asked about the type of cultivation adopted (traditional with the use of pesticides, organic, or mixed).

Subsequently, the farmers completed a GAP questionnaire in checklist format [6]. The instrument consists of 11 blocks, totaling 128 items, covering the following topics: (i) history and management of the rural property; (ii) propagation material; (iii) soil and other substrate management; (iv) fertilization; (v) water management; (vi) crop protection; (vii) presence of animals on the rural property; (viii) hygiene and health; (ix) transportation; (x) waste and pollutant management; (xi) training.

For analytical purposes, the 128 items are grouped into 44 composite indicators based on conceptual similarity and alignment with the IICA framework [6]. Each indicator was derived from a set of related checklist items and classified as compliant (1) or non-compliant (0) according to predefined criteria (e.g., majority rule or presence of critical practices). These indicators were then organized into four dimensions: environmental (12 indicators), food safety/inocuity (12 indicators), worker-related aspects (10 indicators), and economic aspects (10 indicators). Scores for each dimension were calculated as the proportion of compliant indicators normalized to a 0–10 scale. The overall percentage of positive impact was calculated as the total number of compliant indicators divided by the total number of indicators (44), multiplied by 100 [6].

##### Perceptions of Good Practices in Agricultural Management

To understand farmers’ perceptions of GAP, the researchers conducted semi-structured interviews [15,16]. The interview took place after the food collection and application of the sociodemographic and GAP questionnaires. In a quiet location on the property, the farmers answered the following guiding questions:

1. How do you perceive the application of Good Agricultural Practices in food production on your property, from cultivation to delivery to schools?
2. What are the difficulties and facilities you perceive in carrying out tasks that could help improve the quality of the products?

### 2.3. Laboratory Analyses

#### 2.3.1. Microbiological Tests

Samples of fruits and vegetables, soil, and water were subjected to bacteriological tests in triplicate. Petrifilm™ plates were used for this purpose, according to the manufacturer’s instructions (3M do Brasil, Ltda., Sumaré, SP, Brazil) [11]. In the case of water, the COLItest^®^ method preceded the use of Petrifilm. The analysis allowed for the simultaneous evaluation of the presence of total coliforms and *E. coli* as indicators of pathogenic microorganisms [17]. In the case of a positive result, the microorganisms were quantified using Petrifilm EC cont Colif *E. coli*^®^ plates. The microorganisms evaluated and the plates used are described in Table 1.

#### 2.3.2. Parasitological Tests

The presence of enteroparasites in fruits and vegetables, soil, and water was diagnosed using the modified spontaneous sedimentation method, as described by Maldonade and Machado (2017) [12]. The analysis for the detection of developmental forms of parasites followed the guidelines described by Marinho et al. (2017) [18]. The procedure was repeated once.

#### 2.3.3. Feces

The material was processed in the laboratory using the parasitological methods of Rugai, spontaneous sedimentation, and the Erlich Ziehl-Neelsen, as described by Neves (2016) [19]. The analysis followed the methodology described by Maldonade and Machado et al. (2017) and Marinho et al. (2017) [12,18].

### 2.4. Data Analysis

#### 2.4.1. Descriptive Analyses

The results of the microbiological analyses were tabulated in a Microsoft Excel^®^ spreadsheet and compared with parameters established in the literature for each sample type. For fruits and vegetables, the references used are listed in Table 2. For soil samples, the results were compared with the guidelines described in the Embrapa publication [20].

#### 2.4.2. Analysis of the GAP Questionnaire

When responses are entered into the IICA/Embrapa System according to the guidelines, a radar chart is generated, consisting of four color-coded lines: green (environmental), blue (occupational safety), red (food safety), and orange (economic) [6]. The chart is structured around two key points: point 0, located at the center, representing the worst GAP condition, and point 1, on the outer edge, indicating the best condition. The closer the lines are to the center, the more critical the problems in that dimension are. Scores should be interpreted according to the following criteria:

- A score of 0% indicates “No Positive Impact.”
- A score of 100% indicates “Maximum Positive Impact.”
- Scores between 1% and 99% indicate a need to “Apply Environmental Management.” [6].

#### 2.4.3. Content Analysis and Data Integration (Triangulation)

The analysis of the interviews followed the principles of content analysis proposed by Bardin (2016) [25]. Thus, it was developed in three stages: (i) pre-analysis; (ii) exploration of the material; and (iii) treatment of results, inference, and interpretation [25]. With the aid of IraMuTeQ^®^ (Interface de R pour les Analyses Multidimensionnelles de Textes et de Questionnaires) version 0.7 Alpha 2 and R version 3.2.3, the researchers performed Hierarchical Descending Classification (HDC). The HDC analysis correlates text segments based on their respective vocabularies, retrieving each segment and its associations, enabling the statistical grouping of significant words into classes and the qualitative analysis of the data. The text segments that make up each class share similar vocabulary and are distinct from those of other classes [26].

In the stage addressing the treatment and interpretation of results, the data were analyzed based on the quantitative findings. The aim was to understand the content of the interviews in the context of school education, property structure (electricity, basic sanitation, and waste management), results of microbiological and parasitological analyses, and adherence to GAP.

Therefore, the triangulation of quantitative and qualitative data was carried out intentionally, systematically, and in an integrated manner, in accordance with the methodological assumptions of the Mixed Methods Appraisal Tool (MMAT). After analyzing the data separately, respecting the methodological rigor of each approach, the integration itself was carried out, identifying convergences, complementarities, and divergences among the results. In this way, there was an integrated interpretation of the data, seeking to understand the quantitative data in a more in-depth and problematized way grounded in the qualitative evidence, producing meta-inferences.

## 3. Results

### 3.1. Quantitative Data on Properties and Laboratory Analyses

#### 3.1.1. Farmer Profiles and Property Structures

The farmers interviewed (n = 9) were, on average, 48.9 ± 11.0 years old, male (100%), predominantly mixed race (88.8%), and had a medium level of education (66.6%). Furthermore, 55.5% of properties achieve an income from agricultural production between US$200.00 and US$400.00 (Table 3).

Regarding sanitation on the farmers’ properties, its availability proved precarious. Sewage disposal in most farms (77.7%) occurs in septic tanks or filter pits (55.5%). Water supply was predominantly from deep or artesian wells (77.7%), with piped water in the residence (88.8%). Most of the waste generated on the properties is collected by government cleaning services, either through direct collection or via a dumpster (66.6%) (Table 3).

#### 3.1.2. Microbiological Analyses

Laboratory analyses revealed high levels of the investigated microbiological in some samples. Fruits and vegetables, in general, present total coliform levels above the standards established in the consulted references in 100.0% (n = 81) of the samples from the nine properties. For mesophilic aerobic bacteria, the standards were exceeded in 96.3% (n = 78) of the samples collected, and *E. coli* was detected in three properties. No *S. aureus* was detected in any of the vegetable and fruit samples. The presence or absence of bacteria in the soil and water can be verified in Table 3.

#### 3.1.3. Parasitological Analyses

Regarding the presence of parasites in the samples, in all properties visited, at least one food sample collected showed contamination by evolutionary forms of enteroparasites (50.6%; n = 41). Researchers found organisms belonging to the phylum Amoebozoa (*Entamoeba coli* and *Endolimax nana*), the phylum Platyhelminthes (*Hymenolepis nana*), and the phylum Nematoda (*Ascaris* sp., *Toxocara* sp., *Strongyloides*, some *Rhabditoides* and *Filaroides* larvae, as well as eggs and females of other unidentified nematodes (100). *Endolimax nana* eggs were identified in two properties, with one contaminated sample in each. *Hymenolepis nana* eggs were found in only one sample (1.2%), as were *Toxocara* sp. and *Ascaris* sp. eggs, *Strongyloides larvae*, and one female nematode. Among the organisms of the phylum Arthropoda, mites, mosquitoes, and other insects were identified. Fleas and microcrustaceans were also found.

In the soil, researchers identified five parasite species. The most common were nematodes (*Rhabditoides* and *Filaroides larvae*), and the least prevalent was *Trichuris* sp. (egg). Parasitological analyses of the feces of the six farmers revealed the presence of *Entamoeba coli* or *Ascaris lumbricoides* eggs in 33.3% of the farmers.

Upon applying the GAP questionnaire, the properties achieved an average positive impact percentage of 64.0% (SD = 12.0), suggesting partial and fragmented adoption of GAP. The recurring critical areas were “worker hygiene and health” and “waste management and capacity building. The areas with the best relative performance were “soil management,” “transport/marketing,” and “general management”.

**Figure 2:**
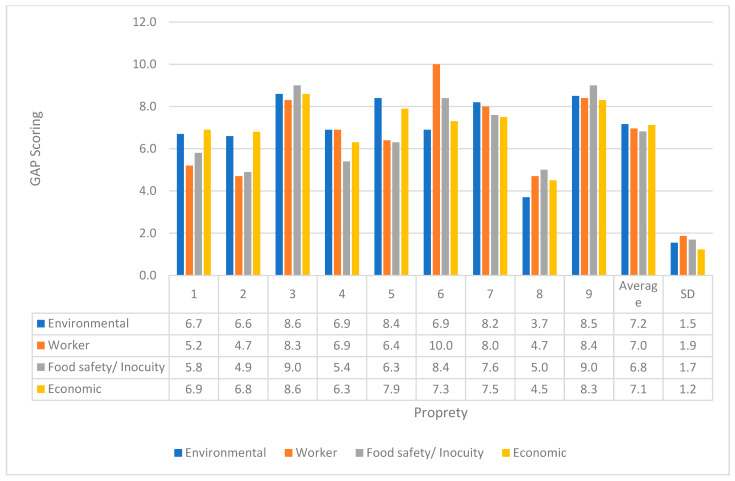
Scoring and overall average of Good Agricultural Practices according to the manual “Good agricultural practices for a more resilient agriculture: Guidelines for guiding producers and governments” of the properties participating in the research, in relation to the environmental, economic, food safety, and worker aspects.

Figure 2 presents the distribution of average GAP scores per property across the four evaluated axes, highlighting heterogeneity within properties and differences between the axes. The “environmental” axis generally shows higher, more homogeneous values, with Property 3 standing out, achieving the highest scores across all axes, indicating better overall GAP performance. In contrast, the “worker” and “food safety/innocuity” axes exhibit greater variability and lower scores on several properties, particularly properties 2, 4, 7, and 8, indicating weaknesses in health, hygiene, and safety. The “economic” axis maintains intermediate values, suggesting productive viability, but, by itself, does not guarantee better sanitary results. The overall average indicates median values of GAP, reinforcing that adoption is partial and uneven across the axes, with more consistent progress in the environmental component and persistent gaps in aspects directly related to food safety and worker health. The radar chart generated for each property is available as Supplementary Materials.

### 3.2. Qualitative Data on Family Farmers’ Perceptions of Good Agricultural Practice Adoption on Their Farms

**Figure 3:**
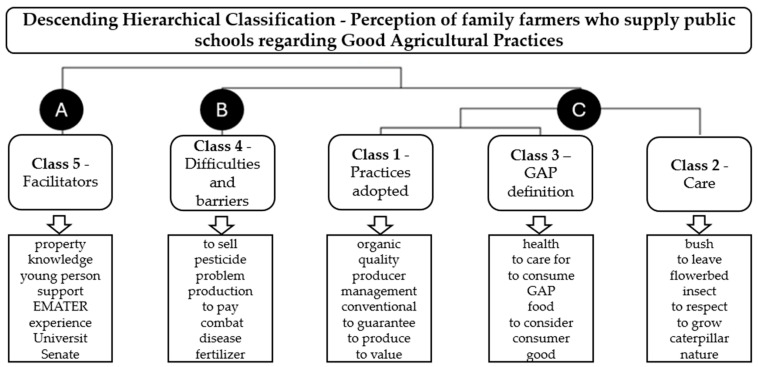
Dendrogram of the lexical analysis of interviews with family farmers participating in the research, with the most frequently used words in the interviews, in descending order.

Based on the interview results, the findings revealed five lexical classes, organized into three broader thematic groups, that express how farmers understand, implement, and face challenges related to GAP. Overall, the discourses indicate a predominantly positive and ethical perception of GAP, strongly associated with organic management, environmental care, and the visual quality of food, but weakly connected to technical criteria of food safety, which helps explain the discrepancy observed between declared perceptions and laboratory findings (Figure 3).

**Class 1—Practices Adopted**

Class 1 consists of arguments related to the practices adopted by farmers. Among the highlighted perceptions are the belief that delivering pest-free products is synonymous with healthy food; that appearance and cleanliness are indicative of a quality product, but are only visible; visible dirt (pests) is emphasized; and that organic farming, for those who practice it, is sufficient to ensure good practices and relates to the producer and how they manage their crop, associating these practices with emotional aspects (care).

Statements:

*“Producing organic products is a responsibility. It’s a commitment the producer must make to produce organically and guarantee the quality of their product. So, on the other hand, the producer needs to rethink this concept and value it.”*

**(F2)**

*“I think it all comes down to knowledge. When a plant is sick, the first thing you notice is that it’s different; it’s turning yellow or not producing properly…”*

**(F7)**

**Class 2—Care Practices**

Regarding care in agricultural activities, the mentions compiled in Class 2 reflect a range of perceptions and practices. Emphasis is placed on the use of sustainable practices, such as natural biological control in organic management, highlighting knowledge about the role of plants and insects in the balance of crops. Farmers also associate hygiene practices and proper management with ensuring product quality. However, they do not seem to perceive the full extent of these aspects. Furthermore, technical assistance is recognized as a valuable resource for addressing more complex challenges, such as combating fungi and pests.

Statements:

*“If we respect nature, we have these beneficial animals that come to try to help. We understand, so the little tinamous walk among the flowerbeds, and if they see a caterpillar, they go there and eat that caterpillar that is damaging the base of the plant”*

**(F2)**

*“You have to be careful not to use anything that could harm insects like bees, or things that help us all.”*

**(F9)**

**Class 3—GAP Definition**

The farmers’ definition of GAP is evident in Class 3 of the analysis. However, they are not addressed as hygiene standards and regulations to ensure food safety. Once again, the aesthetic aspect is evident, and being organic is synonymous with hygiene. Apparently, there is a perception that the only invisible contaminant is a chemical one. It is therefore clear that educating farmers about food safety is urgent.

*“…so we wash it well just with water, only with water, because it’s less harmful. The organic product has a bacteria that is beneficial to our health, you can research it.”*

**(F2)**

*“…so whoever is an organic producer thinks carefully and does Good Agricultural Practices, taking care of themselves, their family, nature, the birds that are there, and the consumer who will consume it in the end.”*

**(F2)**

**Class 4—Difficulties and barriers**

Class 4 (Group B) brings together text segments that address the challenges and barriers faced by farmers, illustrating the answers to guiding question 2. Among the main points mentioned are pests, production targets, labor shortages, high product appearance standards for sale, and product devaluation. Unlike the other classes, the statements defended the use of pesticides, associating them with the production of healthy, visually appealing food. However, the aesthetic aspect was once again highlighted. For them, the product’s appearance is seen as a crucial element in its commercialization and valuation.

Statements:

*“Train the workforce. Because it’s pointless, because every three months we have new employees, at most they stay here, so there’s no point in training them.”*

**(F8)**

*“Even if it’s not all for the food production of a school, it goes to a supermarket shelf, and if that product doesn’t look good, it won’t sell.”*

**(F4)**

**Class 5—Facilitators**

The answers to Guiding Question 3 were compiled in Class 5 (Group A) and address the facilitators of agricultural production. Among them, government agency monitoring stands out, with divided opinions: while some point to the need for greater support, others praise the monitoring received. In addition, farmers mentioned training and education as facilitating elements, as well as closer ties with universities, and undertaking trips to exchange experiences and learn.

*“…we always participate in courses, not only from EMATER, but also from the Senate and SEBRAE. I have already participated in some seminars. I have already made some international trips… we have some experience and knowledge of what is allowed.”*

**(F7)**

*“We have training through EMATER. Through SENAC and SEBRAE, people always come here to provide training… especially for those who work with us, it helps a lot.”*

**(F9)**

### 3.3. Data Triangulation

The triangulation of data derived from interviews with the nine family farmers, the self-administered questionnaire on GAP, the sociodemographic and farm characterization questionnaire, and the microbiological and parasitological analyses of produced vegetables, as well as water, soil, and fecal samples (from six farmers) revealed a consistent mismatch between perception, declared compliance, and measured sanitary risk. From a qualitative perspective, interviews showed that farmers predominantly associate GAP with ethical values, environmental care, organic production, and the visual quality of food, with limited incorporation of “invisible” microbiological and parasitological risks. This positive perception was reinforced by sociodemographic data, indicating a predominance of practical experience, variable levels of formal education, and a strong valuation of empirical knowledge—none of which translated into a higher perception of sanitary risk. In parallel, the GAP questionnaire, analyzed according to the IICA/Embrapa framework [6], classified all farms as having moderate impact, with better performance in the environmental and economic axes and marked weaknesses in blocks related to food safety (innocuity), hygiene, waste management, worker health, and training, indicating partial and fragmented adoption of practices.

The integration of these findings with the laboratory results strengthens the triangulation: despite favorable perceptions and a moderate GAP classification, recurrent microbiological and parasitological contamination was observed in vegetables, soil, and water, along with parasitological positivity in 33.3% of the farmers who underwent fecal testing, indicating occupational exposure and potential feedback into environmental contamination cycles. The widespread presence of contamination indicators in the production environment, contrasted with the low risk perception expressed in interviews, demonstrates that declared GAP compliance and practical experience were insufficient to ensure food safety. Thus, the triangulation confirms that sanitary risk is systemically constructed—encompassing environment, production practices, and human health—and that only an integrated approach, including continuous training, strengthening of the sanitary blocks of GAP, and effective incorporation of a One Health perspective, can align perception, practice, and the safety of foods produced by family farming systems.

**Figure 4:**
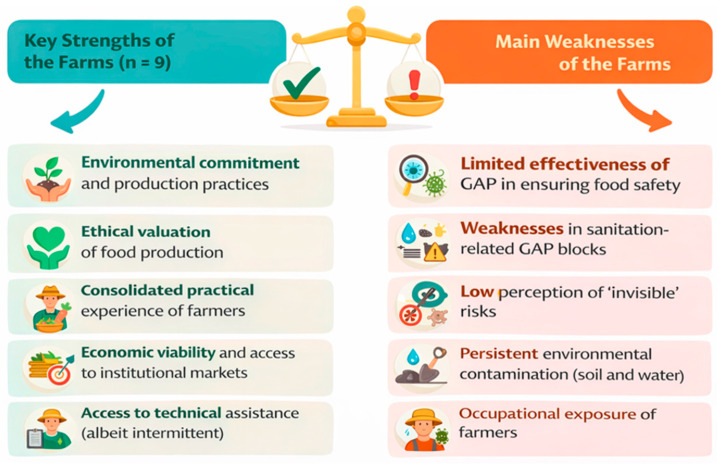
Five main strengths and weaknesses of the evaluated properties and family farmers participating in the research.

Based on the integrated triangulation of qualitative (interviews), quantitative (Good Agricultural Practices questionnaires and sociodemographic profile), and laboratory (food, soil, water, and feces) data, the five main strengths and weaknesses of the evaluated properties are highlighted and illustrated in Figure 4.

## 4. Discussion

The sociodemographic data of the farmers and the conditions of the investigated properties reveal convergence with national data. According to IBGE [27] and IPEA [28], in the Midwest Brazilian Region, family farmers have a predominantly adult age profile, with a higher concentration above 40–45 years, reflecting the aging process of the rural environment observed throughout the country, although with a slightly higher presence of farmers of productive age when compared to Brazilian regions such as the Northeast. This demographic trend is not exclusive to Brazil. Recent studies in other developing countries, such as India and Ethiopia, also highlight an aging farming population and the challenge of generational renewal, which directly impacts the openness to adopting new sanitary technologies and GAP [29,30].

As in the properties visited, the male sex significantly predominates in the formal management of properties in Brazilian territory. However, it diverges from the literature cited, which shows that women play a crucial role in agricultural work and in support activities. Globally, the invisibilization of females in rural management remains a barrier to food safety training, as extension services often target the formal head of household (usually males), thereby missing those who frequently handle and harvest [31]. However, they often lack formal recognition as those responsible for the production unit. The small sample of properties in this study may have prevented verification of this reality, which represents a potential limitation of the study. Regarding color/race, IBGE data indicate that most farmers self-identify as mixed-race, followed by white-race. This pattern resembles the participants in this study and is associated with the history of territorial occupation, agrarian reform settlements, and agricultural expansion fronts in the Midwest region [27,28]. This educational barrier is a well-documented global phenomenon. Research in Portugal and Nigeria confirms that while farmers may have high environmental awareness, translating “invisible” microbiological risks into practice is hindered by low levels of formal education and a lack of tailored technical assistance [32,33].

Furthermore, it is noteworthy that limited educational levels directly impact the ability to access, understand, and apply technical and sanitary standards, such as GAP, especially their more complex components. Regarding income, family farmers in the Midwest region have low to moderate average incomes and are heavily dependent on agricultural activity, frequently supplementing their incomes with rural pensions, income transfer programs, and public policies that support institutional purchases, such as the National School Feeding Program (PNAE).

Regarding the properties’ conditions, as found, electricity was expected, but water supply from alternative sources was inconsistent and not always monitored. The presence of a bathroom and a sewage system is common, according to the literature, but the treatment of effluents is usually inadequate. The persistence of inadequate sewage systems (e.g., non-connected septic pits) and the use of untreated water for irrigation are critical points of failure identified in small-scale farms globally, from Southeast Asia to Latin America, serving as primary drivers for the persistence of pathogens like *E. coli* and *Salmonella* in the production environment [34,35].

Precariousness is also reported in relation to garbage collection, which is predominantly carried out through informal practices, as observed in the properties visited [27,28]. According to Gomes et al. [36], the limitations observed in the infrastructure of the analyzed properties do not primarily stem from individual failures by farmers, but rather from structural and institutional constraints that affect family farming. The historical insufficiency of public investment in rural sanitation, the discontinuity of infrastructure policies, the prioritization of urban areas, and territorial inequalities, even in economically dynamic regions such as the Midwest, restrict access to treated water, adequate sewage disposal, and regular waste collection. Added to this are low average income, difficulties in accessing credit and financing, economic insecurity, and limited technical assistance, which reduce the capacity for private investment in structural improvements. These factors help explain the persistence of precarious sanitation and environmental management conditions on the properties studied [36,37].

The profile of the farmers analyzed reflects a strong emphasis on empirical knowledge and practical experience, evidenced in the interviews, in which farmers associate food quality and safety primarily with organic management, soil care, and the visual appearance of the products. This practical and symbolic capital, while fundamental to the social sustainability of family farming, does not, in itself, guarantee the full incorporation of more complex technical dimensions related to food safety. Furthermore, low income, limited education, and an overload of productive responsibilities limit the capacity for autonomous investment in sanitary improvements, a scenario that directly aligns with the results observed in the analyzed properties [37,38].

The results of the laboratory analyses, showing high levels of parasites and indicator microorganisms, align with international findings where fresh produce from smallholder farms often exceeds safety limits due to environmental exposure. For instance, a recent study in Portugal identified pathogenic bacteria and chemical residues in small-scale farms, highlighting that even when farmers have “good intentions” or follow organic principles, structural gaps in water and soil management lead to contamination [32]. Similarly, research in Ethiopia found that over 80% of vegetable samples were contaminated with bacteria, reinforcing that the pre-harvest environment is a critical nexus of risk [30]. The identification of *Toxocara* sp. suggests the presence of animals, such as dogs and cats, in the planting areas, reinforcing the need for adequate environmental control. This finding is consistent with global reports on the impact of domestic and wild animals as vectors of zoonotic parasites in fresh produce, a risk often underestimated by farmers who perceive their farms as “natural” and, therefore, safe [39]. Furthermore, manual handling during cultivation, associated with inadequate worker hygiene and the absence of sanitary infrastructure, contributes to cross-contamination. Taken together, these factors demonstrate that fruit and vegetable contamination can occur silently and cumulatively even before harvest, reinforcing the need for integrated GAP [40,41,42,43].

The results add to data from the application of the GAP questionnaire, revealing a partial and fragmented adoption of these practices. All properties were classified as having a moderate impact, showing better performance in the environmental and economic areas, but consistent weaknesses in the blocks related to food safety, hygiene, water management, waste, and worker health. This implementation gap, in which environmental sustainability is prioritized over microbiological safety, is a recurring theme in international literature. Smallholder farmers often adopt practices that provide immediate visible benefits (e.g., soil health or pest control) but neglect “invisible” hygiene protocols due to a lack of infrastructure and technical knowledge [5,44]. A study by Kharel et al. [5] indicates that this pattern is recurrent in family farming systems, where environmentally sustainable practices coexist with structural limitations, limited continuous training, and sanitary infrastructure restrictions, exactly as in the scenario studied. Even when farmers demonstrate adherence to sustainable practices, integration into institutional markets, and a positive perception of GAP, these initiatives are not sufficient to overcome structural weaknesses without continuous government support, qualified technical assistance, and adequate financing [37].

Partial adoption directly relates to farmers’ discourse, which often reduces GAP to ethical, environmental, and productive principles. Microbiological and parasitological analyses reinforce this discrepancy: despite the positive perception and declared compliance with GAP, recurrent contamination was observed in food, soil, and water, and parasitological positivity was observed in some of the farmers evaluated. It is also worth noting that, in organic and family production systems, the absence of adequate infrastructure and systematized sanitary practices favors the persistence of biological risks, even when there is intention and commitment to sustainable practices [38].

Finally, the triangulation highlights that the productive environment serves as a central nexus of risk, integrating soil, water, food, and farmer health, while the discourses reveal a low perception of the “invisible” risks posed by microorganisms and parasites. This mismatch between perception and reality is a global challenge. As seen in studies from Thailand to Peru, farmers often equate organic or fresh with safe, ignoring the need for rigorous sanitary controls [35]. Strengthening GAP requires more than formal adherence; it demands a shift in the food safety culture of rural areas, supported by continuous government investment in rural infrastructure and specialized technical assistance [45]. In light of Tanure et al. [46], this scenario must be understood within the broader context of contemporary family farming, marked by structural limitations, dependence on public policies, and the need for social and institutional innovation to sustain its economic and sanitary reproduction. Thus, the results indicate that strengthening GAP requires more than formal adherence. It demands continuous training, qualified technical assistance, improvement of rural infrastructure, and effective integration of human, environmental, and productive health. The convergence among quantitative data, laboratory evidence, and farmers’ narratives reinforces the urgency of intersectoral strategies, aligned with the One Health approach, capable of reducing the gap between perceptions, practices, and real sanitary risk in family farming [46]. The following table summarizes the data triangulation performed (Table 4).

### Study Limitations

The main limitation of this study is the small sample size (nine farms), which is not representative. In addition, only six farmers agreed to collect fecal samples, resulting in limited data that does not allow us to evaluate the real health risks to farmers. In addition, female farmers often lack formal recognition as those responsible for the production unit and the small sample of properties in this study may have prevented verification of this reality. Despite efforts to gain access to family farmers, this was the biggest challenge for the researchers. The difficulty in visiting the properties and obtaining consent to participate in the research made a larger sample unfeasible, thus preventing more robust inferences about the investigated scenario.

Another limitation is the lack of validation of farmers’ responses to the questionnaires against regulatory information from agricultural or health authorities. We understand that the responses may have been limited by the farmer’s knowledge, leading to overestimates or underestimates of lived realities. However, we emphasize that the main aim was to understand farmers’ perceptions of their reality, using laboratory analyses as a benchmark.

## 5. Conclusions

The results of this research reveal important tensions between the potential and challenges faced by participating family farmers. Sociodemographic data indicate producers with strong practical experience, a direct link to the land, and a positive perception of their social and environmental role. These findings converge with the literature that recognizes family farming as a strategic agent of sustainability. However, when analyzing the conditions of the properties and the microbiological and parasitological results, it is observed that inclusion in the PNAE and the declared adoption of GAP are not sufficient to systematically ensure food safety. The precarious sanitary conditions of some properties may contribute to the persistence of environmental and product contamination, associated with weaknesses in the sanitary aspects of GAP. This shows that institutional procurement policies, while fundamental, need to be accompanied by continuous training, qualified technical assistance, and integrated actions for environmental and occupational health. Thus, the findings reinforce the need to strengthen the PNAE as a policy for promoting adequate and healthy food, as this depends not only on market access but also on consolidating intersectoral strategies that align productive sustainability, food safety, and the protection of human health within a One Health approach.

## Figures and Tables

**Table 1:** Microorganisms investigated and culture media used in fruits and vegetables, soil, and water from family farming properties supplying public schools in the Federal District, Brazil.

Microrganism	Culture Medium	Sample		
		F&V	Soil	Water
*Staphylococcus aureus*	3M Petrifilm STX + *S. aureus*^®^	X		
Aerobic mesophiles	Petrifilm AC cont tot bact^®^	X		
Total and thermotolerant coliforms and *Escherichia coli*	Petrifilm EC cont colif *E. coli*^®^	X	X	X
Total coliforms and *Escherichia coli*	COLItest^®^			X

**Table 2:** Microbiological standards for food used to verify suitability in family farming properties that supply public schools in the Federal District, Brazil.

Microorganism	Reference Values	Reference
Total coliforms	10^2^ CFU/g.	RDC nº 12, 2001 [21]
*E. coli*	10^3^ CFU/g.	IN nº 161, 2022 [22]
Aerobic mesophiles	10^5^ CFU/g.	Santos, 2021 [23]
*S. aureus*	10^6^ CFU/g.	Sant’ana & Azeredo, 2005 [24]

**Table 3:** Sociodemographic description of the farmers, general data of the investigated properties including GAP adherence, and results of parasitological and microbiological analyses of fruit and vegetable, soil, and water samples collected on family farming properties that supply public schools in the Federal District, Brazil, and of farmers’ feces.

	Property		1	2	3	4	5	6	7	8	9
Owner profile	Gender		Male	Male	Male	Male	Male	Male	Male	Male	Male
	Age (years)		46	49	43	60	54	37	44	35	72
	Self-identified skin colour/race category		Black	Brown (‘pardo’)	Brown (‘pardo’)	Brown (‘pardo’)	Brown (‘pardo’)	Brown (‘pardo’)	Brown (‘pardo’)	Brown (‘pardo’)	Brown (‘pardo’)
	Educational level		Incomplete University degree	Incomplete elementary school	Complete secondary school	Complete elementary school	Incomplete elementary school	Complete secondary school	Incomplete University degree	Complete University degree	Complete secondary school
	Financial returns from agricultural production (U$)		200–400	401–600	200–400	601–1000	1001–2000	401–600	1001–2000	1001–2000	2001–4000
Property Features	Electrical energy		Yes	Yes	Yes	Yes	Yes	Yes	Yes	Yes	Yes
	Water supply		Shallow well, water table well, or hand-dug well	Deep well or artesian well	Public water supply network	Deep well or artesian well	Deep well or artesian well	Deep well or artesian well	Deep well or artesian well	Deep well or artesian well	Deep well or artesian well
	Piped water available		Yes	Yes	Yes	No	Yes	Yes	Yes	Yes	Yes
	Number of bathrooms		1	2	2	2	3	2	7	2	5
	Bathroom shared with other households		No	No	Yes	Yes	Yes	No	Yes	No	Yes
	Type of sanitary waste disposal		Septic tank or filter tank—not connected to the sewer system	Septic tank or filter tank—not connected to the sewer system	Septic tank or filter tank—not connected to the sewer system	Septic tank or filter tank—not connected to the sewer system	Septic tank or filter tank—not connected to the sewer system	Rudimentary pit or hole	Septic tank or filter tank—not connected to the sewer system	Ecological septic tank	Septic tank or filter tank—not connected to the sewer system
	Method of waste disposal		Burned on the property	Collected on the property by cleaning service	Burned on the property	Collected on the property by cleaning service	Burned on the property	Deposited in a public cleaning service dumpster	Deposited in a public cleaning service dumpster	Deposited in a public cleaning service dumpster	Deposited in a public cleaning service dumpster
Presence of microorganisms indicative of possible contamination by pathogens	Fruits and vegetables samples (n = 81)	Total coliforms (>10^2^ CFU/g)—100% (n = 81)	100.0% (n = 9)	100.0% (n = 9)	100.0% (n = 9)	100.0% (n = 9)	100.0% (n = 9)	100.0% (n = 9)	100.0% (n = 9)	100.0% (n = 9)	100.0% (n = 9)
		*E. coli* (>10^3^ UFC/g)—3.7% (n = 3)	0.0% (n = 0)	0.0% (n = 0)	11.1% (n = 1)	11.1% (n = 1)	11.1% (n = 1)	0.0% (n = 0)	0.0% (n = 0)	0.0% (n = 0)	0.0% (n = 0)
		Mesophilic aerobes (>10^5^ CFU/g)—96.3% (n = 78)	100.0% (n = 9)	100.0% (n = 9)	100.0% (n = 9)	100.0% (n = 9)	88.9% (n = 8)	100.0% (n = 9)	100.0% (n = 9)	88.9% (n = 8)	88.9% (n = 8)
		*S. aureus* (>10^6^ CFU/g)—0.0% (n = 0)	0.0% (n = 0)	0.0% (n = 0)	0.0% (n = 0)	0.0% (n = 0)	0.0% (n = 0)	0.0% (n = 0)	0.0% (n = 0)	0.0% (n = 0)	0.0% (n = 0)
	Surface soil samples (n = 45)	Total coliforms—86.7% (n = 39)	60.0% (n = 3)	60.0% (n = 3)	100.0% (n = 5)	80.0% (n = 4)	100.0% (n = 5)	100.0% (n = 5)	100.0% (n = 5)	100.0% (n = 5)	80.0% (n = 4)
		*E. coli*—20.0% (n = 9)	0.0% (n = 0)	0.0% (n = 0)	40.0% (n = 2)	0.0% (n = 0)	20.0% (n = 1)	0.0% (n = 0)	60.0% (n = 3)	60.0% (n = 3)	0.0% (n = 0)
	Deep soil samples (n = 45)	Total coliforms—86.7% (n = 39)	60.0% (n = 3)	60.0% (n = 3)	100.0% (n = 5)	100.0% (n = 5)	100.0% (n = 5)	100.0% (n = 5)	100.0% (n = 5)	100.0% (n = 5)	60.0% (n = 3)
		*E. coli*—22.2% (n = 10)	0.0% (n = 0)	0.0% (n = 0)	20.0% (n = 1)	0.0% (n = 0)	40.0% (n = 2)	0.0% (n = 0)	80.0% (n = 4)	20.0% (n = 1)	40.0% (n = 2)
	Water sample (n = 18)	Total coliforms—83.3% (n = 15)	No	No	Yes	Yes	No	No	Yes	Yes	Yes
		*E. coli*—33.3% (n = 6)	No	No	Yes	Yes	No	No	No	No	No
Presence of parasites or their evolutionary forms	Fruits and vegetables samples (n = 81)—50.6% (n = 41)		100.0% (n = 9)	44.4% (n = 4)	22.2% (n = 2)	11.1% (n = 1)	22.2% (n = 2)	33.3% (n = 3)	88.9% (n = 8)	100.0% (n = 9)	55.6% (n = 5)
	Soil sample (n = 90)	Surface soil samples (n = 45)—62.2% (n = 28)	60.0% (n = 3)	80.0% (n = 4)	80.0% (n = 4)	100.0% (n = 5)	100.0% (n = 5)	0.0% (n = 0)	20.0% (n = 1)	40.0% (n = 2)	80.0% (n = 4)
		Deep soil samples (n = 45)—64.4% (n = 29)	40.0% (n = 2)	100.0% (n = 5)	60.0% (n = 3)	80.0% (n = 4)	80.0% (n = 4)	40.0% (n = 2)	60.0% (n = 3)	60.0% (n = 3)	60.0% (n = 3)
	Water sample		Negative	Negative	Negative	Negative	Negative	Negative	Negative	Negative	Negative
	Stool samples		Negative	Negative	*Entamoeba coli* cysts (4)	*Entamoeba coli* cysts (+40)	*Ascaris lumbricoides* egg (1)	Unrealized	Negative	Unrealized	Unrealized
Technical guidance on GAP and the assessment results	Receives technical guidance		Yes	Yes	Yes	Yes	No	Yes	Yes	Yes	Yes
	Source of guidance		Government	Government	Government	Government and private planning companies	Government, cooperative and others	Government	Government and others	Government	Government and others
	Method for obtaining technical information		Technical meetings/Seminars	Technical meetings/Seminars	Internet	Others	No source	Internet and others	Internet	Internet	Internet/Technical meetings/Seminars
	Overall GAP Positive Impact (%)		55.8	52.37	78.7	57.9	66.0	74.4	71.2	40.6	77.7
Fertilization and cultivation	Type of fertilization		Chemical	Organic	Chemical and organic	Chemical and organic	Chemical and organic	Chemical and organic	Organic	Organic	Organic
	Use of pesticides		Yes	No	No	No	Yes	No	No	No	No
	Organic farming		No	Yes	Yes	No	No	Yes	Yes	Yes	Yes

**Table 4:** Synthesized triangulation of data from research conducted with family farmers who supply public schools in the Federal District—Brazil.

Analytical Dimension/GAP Axis	Quantitative Findings (GAP Assessment)	Qualitative Findings (Farmers’ Perceptions)	Laboratory Evidence	Integrated Interpretation (Triangulation)
**Farm management and production practices**	Satisfactory compliance (>0.5) in farm history, crop management, propagation material, and transportation	GAP primarily understood as good crop management and compliance with market requirements	Persistent contamination in food, soil, and water	Production-oriented GAPs are prioritized, but do not adequately address sanitary hazards
**Environmental management**	High mean score (7.16), indicating good reported performance	Emphasis on organic fertilization, crop rotation, soil cover, and biodiversity protection	Universal presence of total coliforms in soil	Environmentally sustainable practices alone are insufficient to ensure microbiological safety
**Water management**	Generally satisfactory compliance	Water considered safe based on visual clarity and well source	Detection of total coliforms and *E. coli* in irrigation water	Visual assessment masks microbiological risk, undermining food safety
**Food safety (innocuity)**	88.8% of farms non-compliant; major failures in hygiene and sanitation	GAP equated with washing produce and visual cleanliness; organic food perceived as inherently safe	Total coliforms in 100% of food samples; *E. coli* and parasites detected	Core GAP objective—food safety—is not achieved despite positive self-perception
**Waste management and contaminant control**	Low compliance	Limited recognition of waste as a contamination source	Parasitic forms detected in soil and food	Poor waste management sustains environmental reservoirs of contamination
**Worker health and safety**	Irregular compliance; no systematic health monitoring	Farmers rarely perceive themselves as contamination vectors	*E. coli* and parasites detected in farmers’ feces	Farmers represent an underrecognized link in the contamination chain
**Training and technical assistance**	One of the lowest compliance scores	Training perceived as sporadic and undermined by labor turnover	Recurrent sanitary failures despite reported guidance	Lack of continuous capacity-building weakens GAP effectiveness
**Economic conditions**	Relatively high mean score (7.11), with wide variability	Infrastructure deficits and low economic valuation of organic products reported	Sanitary deficiencies more evident in structurally limited farms	Economic constraints drive selective GAP adoption, prioritizing production over safety
**Overall impact level**	Moderate impact (63.9%); recommendation to apply environmental management	Recognition of need for improvement, but limited systemic understanding	Persistent microbiological and parasitological contamination	GAP adoption is fragmented and non-systemic

## Data Availability

The original contributions presented in the study are included in the article/Supplementary Material, further inquiries can be directed to the corresponding author.

## Supplementary Materials

The following supporting information can be downloaded at: https://www.mdpi.com/article/10.3390/foods15071225/s1, Supplementary Figures: Multidimensional Assessment of Good Agricultural Practices Indicators (Integral Approach).

## Abbreviations

EMBRAPA	Brazilian Agricultural Research Corporation
EMATER	Technical Assistance and Rural Extension Company of the Federal District
FBD	Foodborne diseases
FD	Federal District
GAP	Good Agricultural Practices
HRAF	Human Right to Adequate Food
IBGE	Brazilian Institute of Geography and Statistics
IPEA	Institute of Applied Economic Research
MMAT	Mixed Methods Appraisal Tool
PNAE	National School Feeding Program
SDG	Sustainable Development Goal
