# Supplementary figures and images for: Perception and Adoption of Good Agricultural Practices Among Family Farmers Supplying Fruits and Vegetables to Brazil’s School Feeding Program—A Mix-Method Study in the Federal District
Source: Foods. 2026 Apr 3;15(7):1225. doi: 10.3390/foods15071225 (PMC13073351; doi:10.3390/foods15071225)

# Property 1

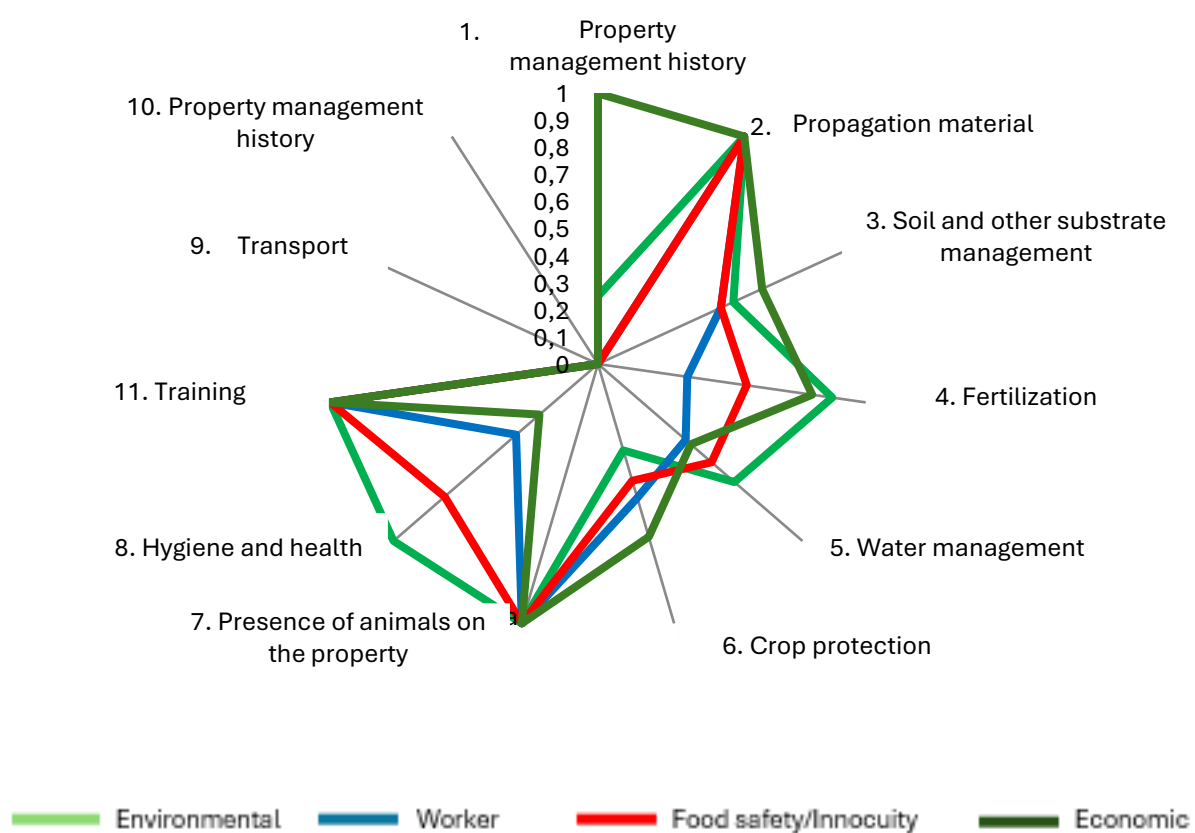

# Property 2

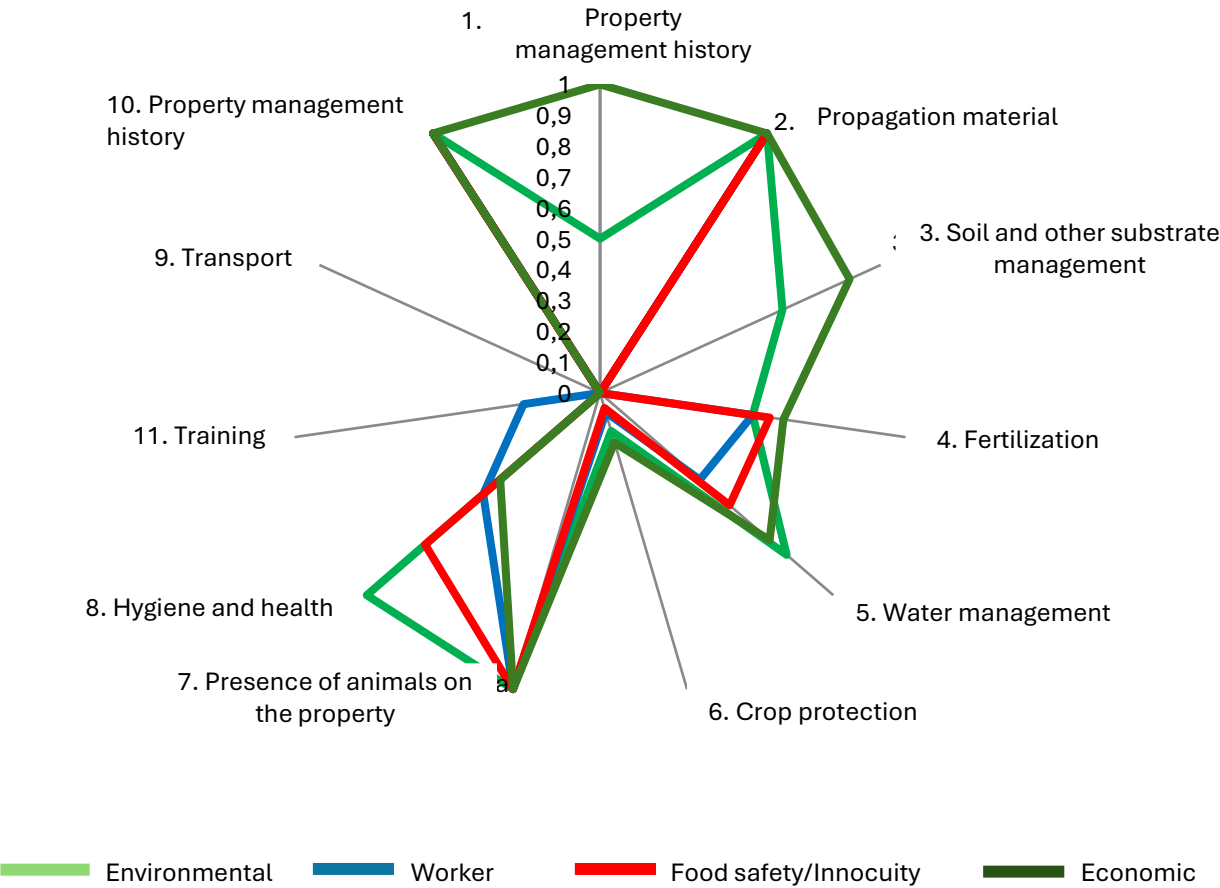

# Property 3

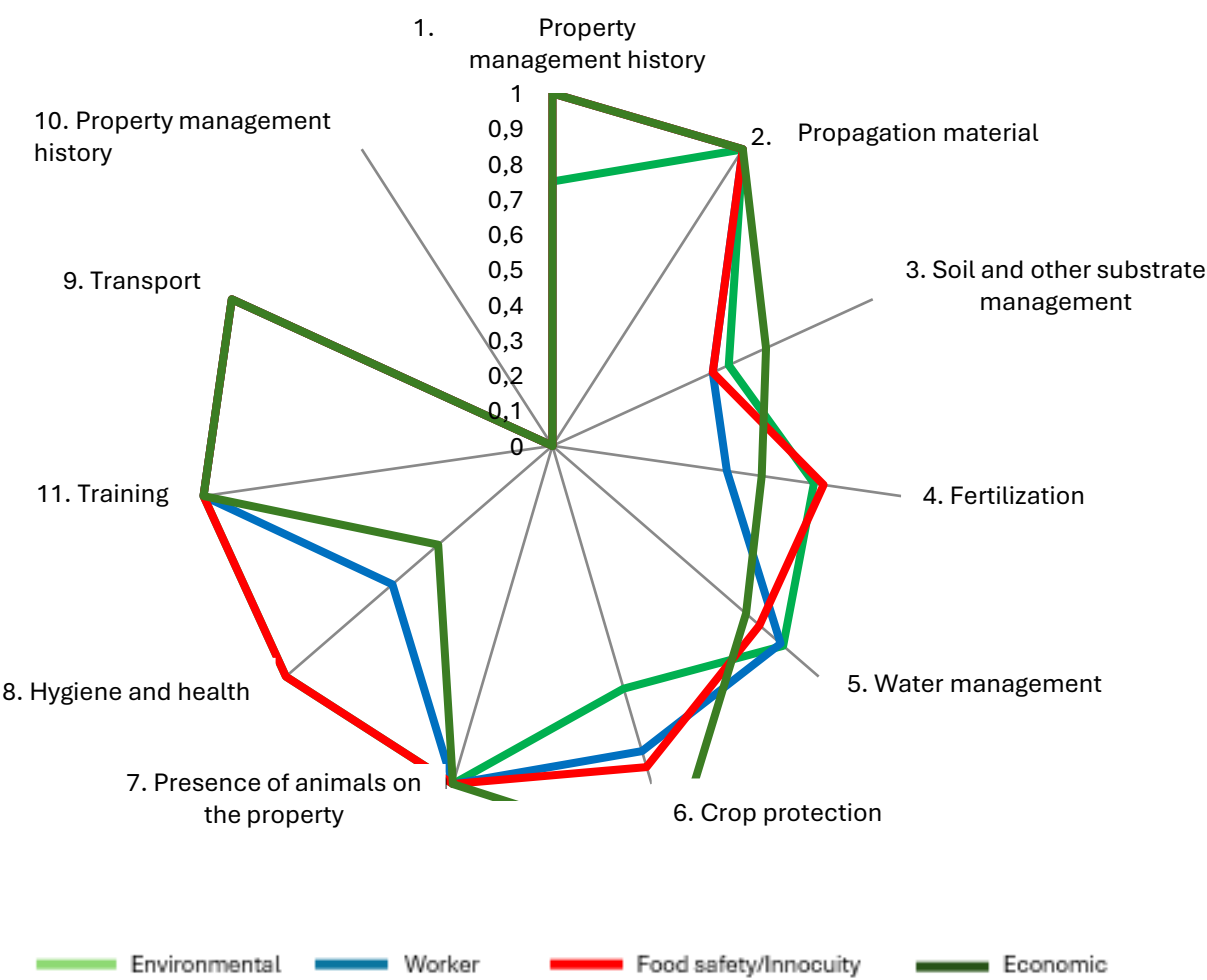

# Property 4

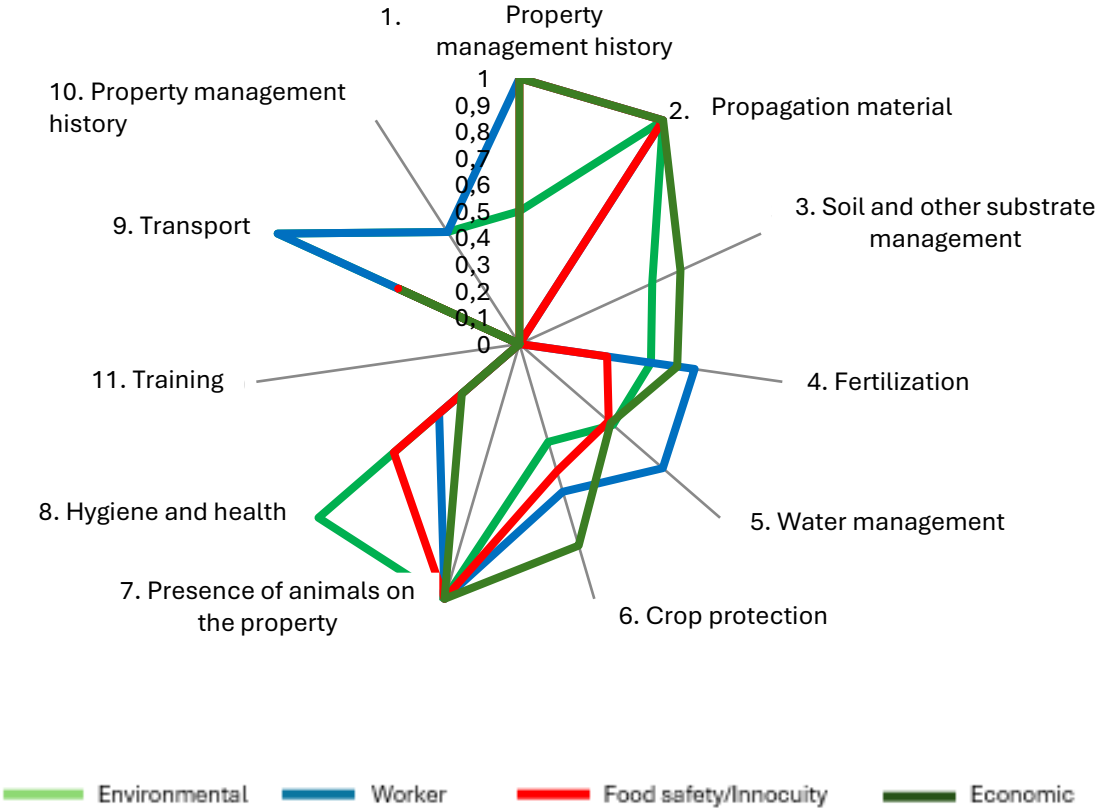

# Property 5

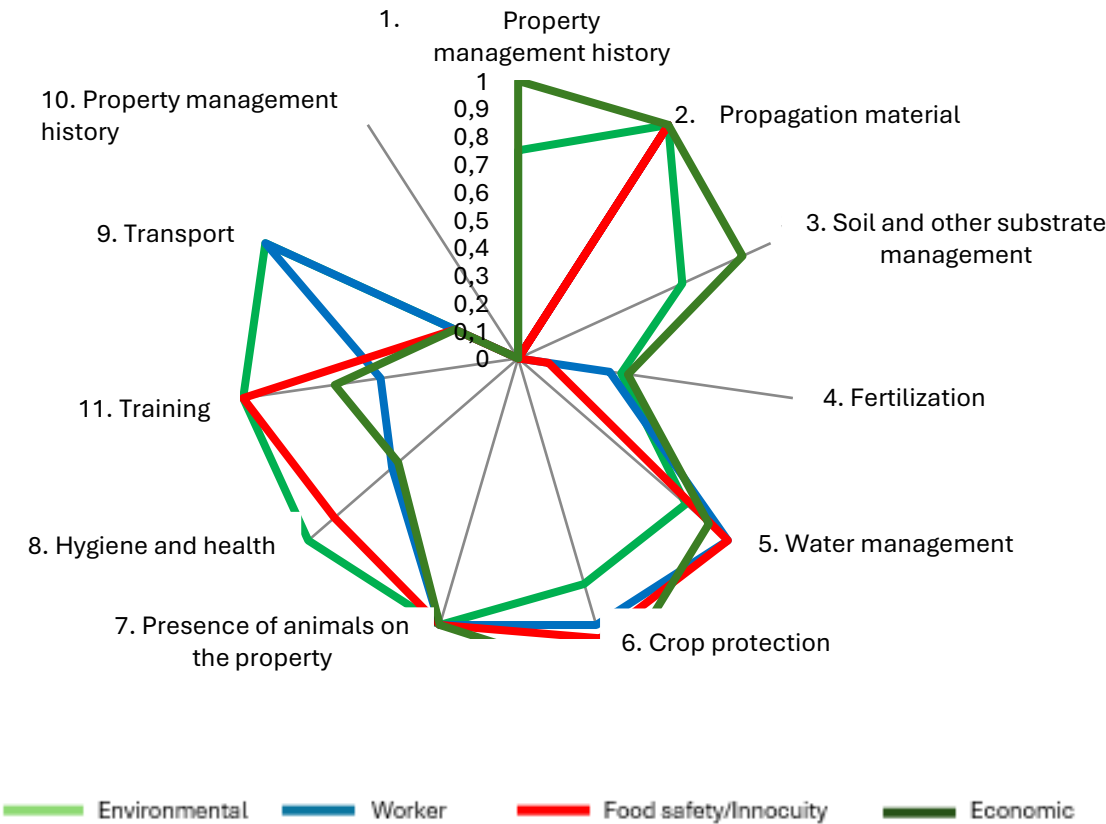

# Property 6

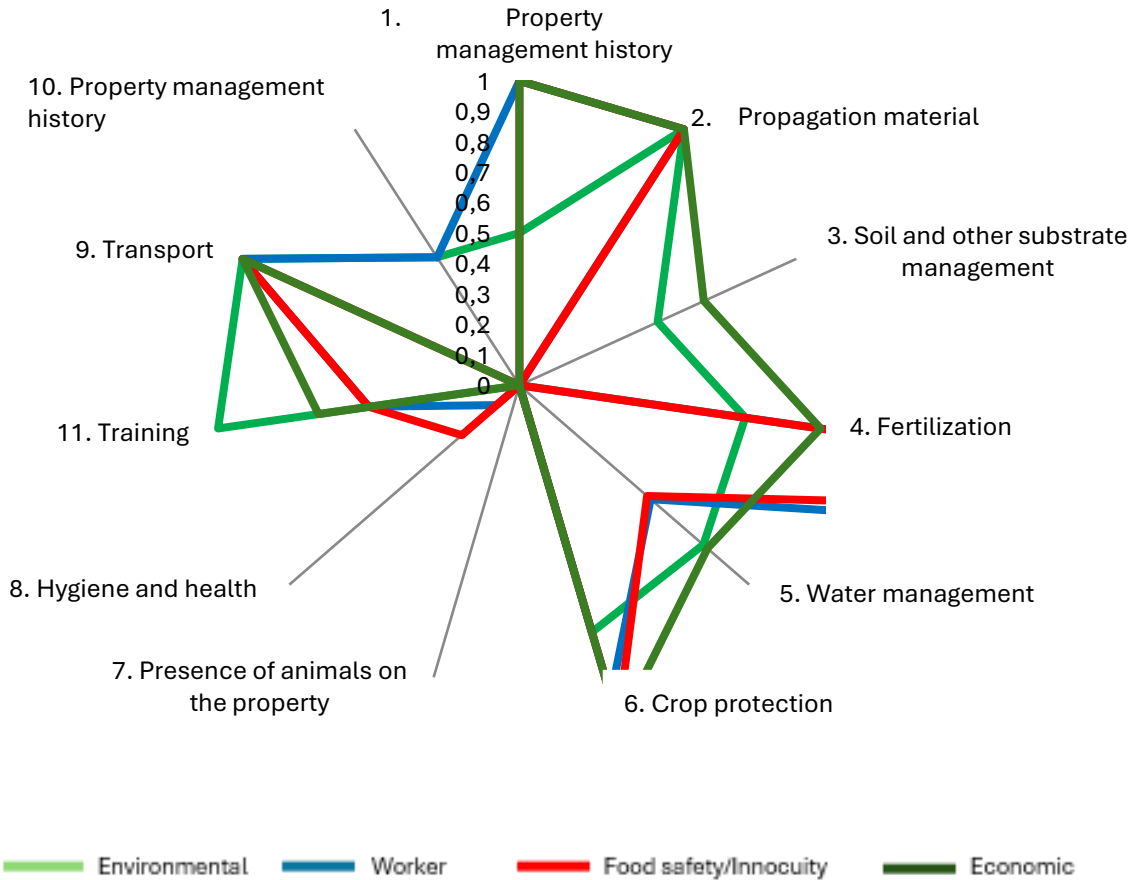

## Property 7

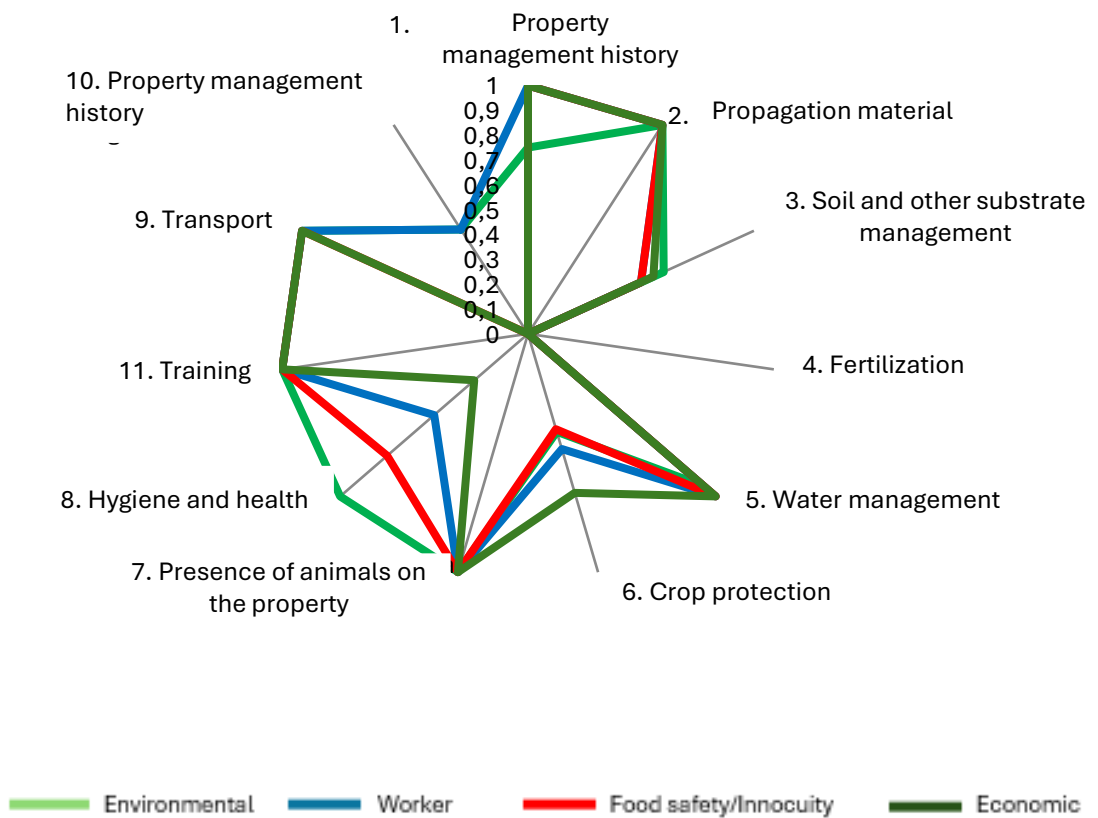

# Property 8

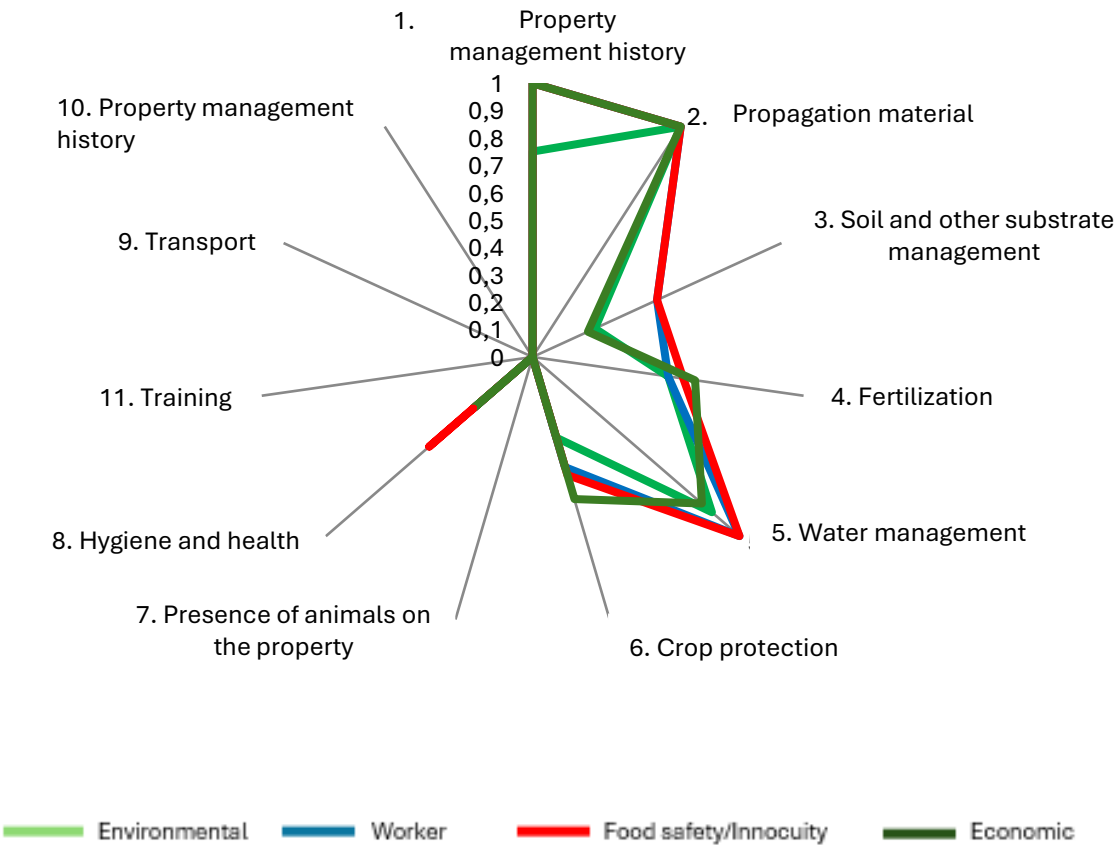

# Property 9

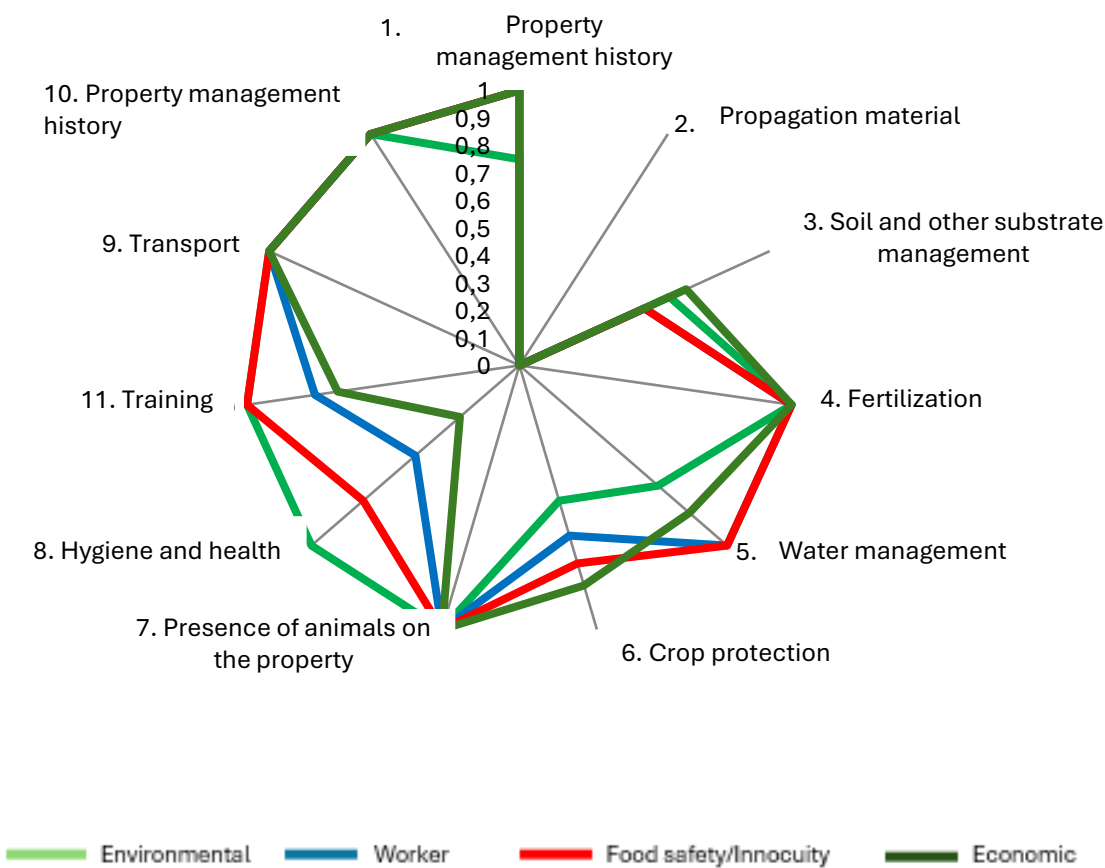

Supplement: Supplementary file 1 [file foods-15-01225-s001.zip › foods-4207546-supplementary.pdf]
